# Surgical Textbook Outcomes in the Era of Neoadjuvant Systemic Treatment for Skin Cancers

**DOI:** 10.3390/jcm13226922

**Published:** 2024-11-17

**Authors:** Piotr Jan Błoński, Piotr Rutkowski, Krzysztof Ostaszewski, Maria Krotewicz, Anna M. Czarnecka

**Affiliations:** 1 Department of Soft Tissue/Bone Sarcoma and Melanoma, National Research Institute of Oncology, 02-781 Warsaw, Poland; s085001@student.wum.edu.pl (P.J.B.); piotr.rutkowski@nio.gov.pl (P.R.); krzysztof.ostaszewski@nio.gov.pl (K.O.); maria.krotewicz@nio.gov.pl (M.K.); 2 Faculty of Medicine, Medical University of Warsaw, 02-106 Warsaw, Poland; 3 Doctoral School of Molecular Medicine, Medical University of Lodz, 90-647 Lodz, Poland

**Keywords:** textbook outcome, neoadjuvant therapy, skin cancer, melanoma

## Abstract

Recent years have brought new, highly effective systemic treatments to clinical practice, which can be used to treat patients with locally advanced or metastatic skin cancers. Using these regimens in neoadjuvant strategy influences surgical treatment by facilitating surgical resection, avoiding extensive resections with complex reconstructions and even omitting surgery in some cases. Integrating systemic therapy with surgery is ongoing and requires novel quality measures of surgical treatment to capture the clinical benefits of multidisciplinary strategies better. The Textbook Outcome (TO) is a novel measure of surgical quality, which captures the short-term outcomes of surgery and reflects long-term survival. Textbook Outcomes match a particular type of surgery, are intuitive to interpret, and may be widely applied in surgical oncology and general surgery. Therefore, this review aims to describe recent findings on neoadjuvant skin cancer treatment and their implications for surgical proceedings in the context of Textbook Outcomes.

## 1. Introduction

Innovative and highly efficient systemic treatments, immune checkpoint inhibitors (ICI) and targeted therapy (TT) for patients with advanced (unresectable and/or metastatic) skin malignancies have shown impressive clinical activity and have significantly improved survival outcomes in these patients [1,2]. They have also laid the groundwork for integrating those efficient systemic therapies with surgery, to maximize the clinical benefit for patients with a resectable disease. The Textbook Outcome (TO) is a novel measure of surgical quality, designed to capture the short-term results of surgery, and reflects long-term survival. We have searched PubMed, Scopus and Google Scholar to identify studies assessing the TO in patients undergoing neoadjuvant treatment for skin malignancies (melanoma and non-melanoma skin cancers) to include those in this review. The search strategy included terms such as (“Melanoma” OR “Skin Cancer”) AND “Textbook Outcome” OR “Neoadjuvant therapy”, then references from the relevant publications were used to identify further studies. The clinical trials were identified using the Clinicaltrials.gov database.

## 2. Integration of Surgery with Systemic Treatment

### 2.1. Cutaneous Melanoma

In patients with resectable melanoma, novel systemic regimens (ICI and TT) were first introduced as palliative, and later as an adjuvant therapy, and together with surgery, they constitute a standard of care in this group of patients today [3,4,5,6]. However, it was proposed that the administration of ICIs prior to surgical resection can result in a better antitumor response of the immune system (as the presence of the tumor stimulates the T cells, which, at the same time, are not suppressed, due to immune checkpoint blockage) and, consequently, in the successful eradication of micrometastases that would remain after curative-intent resection, preventing a relapse [7]. Clinical observations confirmed the validity of these findings, supporting the evidence that the efficacy of the ICIs in the neoadjuvant strategy meaningfully exceeds the adjuvant setting [8,9,10]. The most recent findings are those of the NADINA phase 3 clinical trial, during which the risk ratio for progression, recurrence or death was 0.32 (99.9% CI: 0.15–0.66) in favor of the neoadjuvant strategy—a robust confirmation of the superiority of this approach [9]. Furthermore, the pathological response to preoperative systemic therapy, which is assessed in the surgical sample and is classified depending on the number of viable melanoma cells, turned out to be a reliable prognostic biomarker for event-free survival, earning status as a surrogate biomarker for survival outcomes [9,10,11]. To illustrate this, among patients from the NADINA trial, 59% had a major pathological response, which resulted in 95% estimated one-year recurrence-free survival (RFS), whereas in the group of patients with pathological nonresponse (26% of all patients), the one-year RFS was merely 57% [9]. Another 8% of the intention-to-treat population had pathological partial response, which produced intermediate survival outcomes—one-year RFS of 76%—while the remaining patients were not evaluable. This striking difference in survival outcomes between pathological responders and non-responders corroborates the utility of the pathological response as a biomarker for risk stratification, adjuvant treatment personalization, and even—what will be discussed later—the extent of the curative-intent surgery [9,12].

**Figure 1:**
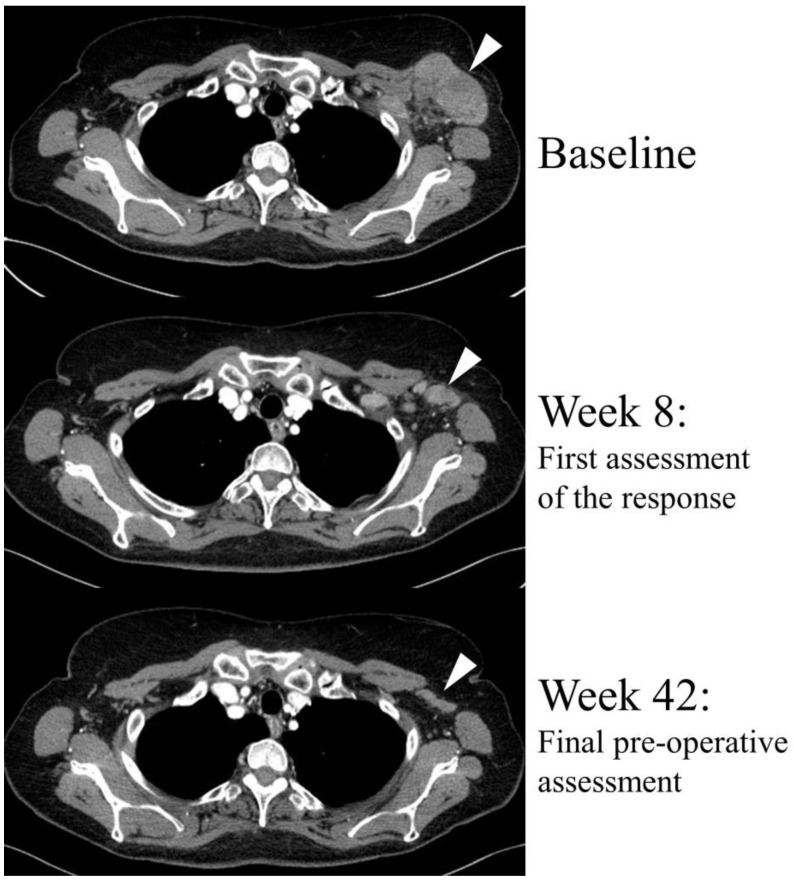
Radiological objective response to the neoadjuvant systemic therapy with dabrafenib plus trametinib (BRAF and MEK inhibitors) in a *BRAF*-mutated, borderline resectable melanoma patient. The response was observed during the first assessment, and further treatment facilitated the surgery. R0 margins were achieved and <10% viable melanoma cells were found in surgical specimen (near-complete pathological response). The target lesion is indicated by white arrows.

The BRAF/MEK-targeted therapy, an alternative to the ICIs, which is available for patients with *BRAF*-mutated tumors, has also been tested in the neoadjuvant setting. Two phase 2 clinical trials showed a satisfactory overall radiological response rate of 64% (an illustration of radiological response to BRAF/MEK-targeted therapy can be seen in Figure 1) and a complete pathological response rate of 47% [10,13,14]. However, unlike immunotherapy, BRAF/MEK-targeted neoadjuvant therapy did not translate into long-term disease-free survival outcomes. A recent pooled analysis revealed that despite the high rates of melanoma-free survival during the first year of follow-up, the responses to TT were not durable and the relapse-free survival deteriorated over the time, whereas ICIs produced long-lasting responses [10,15]. Importantly, these observations regarding TT have also been confirmed in the patients treated in the real-world conditions [16,17]. However, the most severe drawback of neoadjuvant TT is that when relapse occurs, it is more likely to occur within the brain [10,17]. This is in contrast to immunotherapy, which does not exhibit this pattern of recurrence [10].

The combination of neoadjuvant ICI and TT has also been studied. A recent phase 2 clinical trial NeoTrio has investigated three neoadjuvant strategies: two cycles of sole pembrolizumab, sequential therapy with dabrafenib plus trametinib (BRAF plus MEK inhibitors) for one week with subsequent two cycles of pembrolizumab or in the third arm, a concurrent therapy with those regimens [18]. The highest rate of complete pathological responses was seen with concurrent therapy—80%—but those patients have also most often experienced grade 3 or higher adverse events. Importantly, despite numerically higher rates of major pathological responses observed with the addition of TT, the durability of those was worse than with sole ICI, thus putting the long-term efficacy of the triple neoadjuvant strategy in question. Ultimately, the authors have concluded that TT should not be combined with ICI for the neoadjuvant treatment in light of the trial outcome, due to the increase in toxicity and possible deterioration of long-term survival prognosis [18]. Another study investigating the combination of TT and ICI was the NeoACTIVATE trial, which has demonstrated a significant number of pathological responses, but survival outcomes have not been reported yet, due to immature data [19]. Recent clinical trials, investigating neoadjuvant strategies, have been reviewed elsewhere [20,21].

### 2.2. Basal Cell Carcinoma

For patients with basal cell carcinoma of the skin (the most common of all skin cancers), new treatment options have shown activity in locally advanced or metastatic settings, such as targeted therapy with sonidegib or vismodegib (inhibitors of the SHH pathway), and in second-line treatment, such as immunotherapy with cemiplimab (anti-PD-1) [2]. Neoadjuvant TT with vismodegib has been successfully studied in BCC patients and will be described later. ICI neoadjuvant therapy in locally advanced patients with BCC has not been examined so far in a large study; however, preliminary results of an ongoing phase 1b clinical trial with neoadjuvant pembrolizumab (anti-PD-1) have been reported. Of the 13 patients enrolled in the study, two dropped out and 11 underwent surgery after neoadjuvant treatment [22]. Among these 11 patients, three had a complete pathological response. After a median follow-up of 17.6 months, none of the patients experienced relapse [22]. Although the sample size is very small, the preliminary results of this trial show a promising efficacy of pembrolizumab in this setting. These data also suggest that ICIs will probably also affect the neoadjuvant treatment of locally advanced BCC patients.

### 2.3. Cutaneous Squamous Cell Carcinoma

In patients with cutaneous squamous cell carcinoma (CSCC), the second most common type of non-melanoma skin cancer, neoadjuvant immune checkpoint blockage has also been shown to be safe and efficient. In a single-arm phase 2 study, which enrolled patients with high-risk locally advanced but still resectable CSCC, cemiplimab (anti-PD-1 antibody) was tested in a neoadjuvant setting [23,24]. The regimen was well tolerated and yielded a high complete pathological response rate—51% [23]. Similarly to observations made in melanoma patients, the achievement of a complete pathological response to ICIs in CSCC patients resulted in durable disease-free survival [23,24,25]. Namely, the recurrence of the cancer has not been observed in any of the patients with complete pathological response (with the only event-free survival events being attributed to death unrelated to the CSCC or study treatment). Real-world evidence has also confirmed the activity of neoadjuvant ICIs in patients with resectable high-risk CSCC, as it was reported by Kim et al. [26]. Notably, within their study cohort, one-third of patients had a history of hematologic malignancy; most of them were treated for recurrent CSCC (20/27—74.1%), and the median age in the entire cohort was 72 years. The observed rate of complete pathological responses was 36.8%—inferior to those observed in the clinical trial, but still reflecting the clinically significant activity of neoadjuvant ICIs in patients with CSCC under real-world conditions—with an emphasis on the frequent comorbidities in this group of patients [26].

## 3. Textbook Outcomes as a Comprehensive Quality Measure

Results of the surgical treatment can be described by reporting some measures of interest, for example: the rate of R0-margin resections, the rate of complications or the number of patients requiring readmission, all given individually. This can provide us with specific data on the safety of the procedure or likelihood of R0-margin resection. Still, the general outcome of the surgery will be missed, because knowing only the separate factors, we will not be able to combine them into a single image of a particular clinical situation.

**Figure 2:**
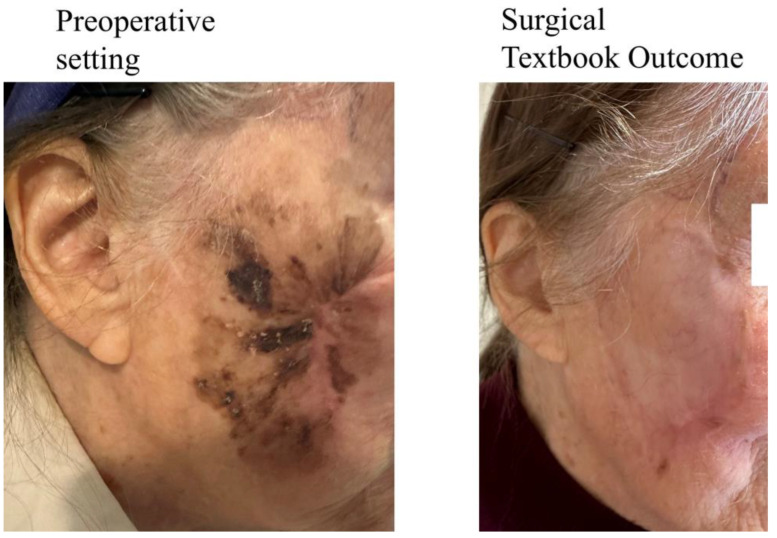
Surgical Textbook Outcome achieved in patient with locally advanced melanoma of the face.

In contrast, if only one comprehensive outcome measure is reported, it could more accurately and informatively reflect the actual level of therapy success and morbidity. Therefore, the idea of putting all individual parameters into one composite measure has been proposed, naming it “Textbook Outcome”. The definition of a Textbook Outcome (TO) includes a list of specific criteria, for example: R0-margins and lack of serious complications, which—pooled together—represent an ideal outcome of the surgery. In other words, a TO represents a state when “everything goes well” in the process of surgical treatment. For illustration, an example of a TO in a patient with locally advanced melanoma of the face is shown in Figure 2. The main principle of the TO is that specific criteria (which may vary between distinct definitions) are required for the achievement of the TO. If they are all met simultaneously, the TO is attained. Otherwise, if any of the criteria has not been met, there is no TO.

The strength of the TO over other composite quality measures is its ability to easily adapt to any procedure, as the definition of a TO is based on expert opinion [27]. This allows for quick implementation of the TO in any field of surgery. Furthermore, the TO is informative for patients, providing them with a straightforward probability of a favorable course of surgical treatment [28]. This makes the TO easy to communicate. Furthermore, despite capturing mainly short-term results, a TO also has prognostic value, as it translates into long-term survival outcomes. As it was shown in the context of several different cancers, patients who achieve TOs have a favorable prognosis over those who do not achieve TOs [29,30,31,32]. Also, the TO is an objective measure, and therefore it can be used for benchmarking [33].

On the other hand, TOs have been criticized for assigning equal weights to all components (i.e., all components of TOs are treated equally important) [27,34]. Therefore, if an exemplary definition of a TO includes, among others, readmission and mortality, the ultimate result of the TO will not differ between the situation in which the patient dies or when they are readmitted and then recover; in both situations the TO will not be achieved. Therefore, to provide transparent reporting of a TO, presenting information on all individual components (criteria) is mandatory.

**Figure 3:**
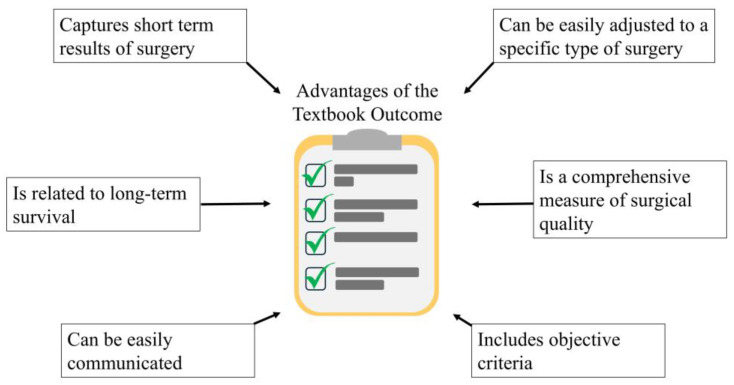
Advantages of the Textbook Outcome in the context of surgical oncology.

In summary, the TO represents an objective, simplistic, intuitive surgical outcome measure, which is easily adjustable to different kinds of surgical procedures, and the selection of the criteria is based upon the experts’ opinion. The TO captures short-term surgical quality and also, it gives prognosis for long-term survival outcomes. The applications of a TO include comprehensive assessment of surgical and oncological quality, prognosis for survival outcomes and straightforward communicating of the expected outcome of surgery to the patient (Figure 3).

## 4. Components of the Textbook Outcome

To define an informative and reliable outcome measure, the TO must cover criteria that are objective and crucial in a specific clinical situation. Thus, there is a great variability of TO definitions across different indications, types of procedures, and even across different studies referring to identical clinical situations. For example, in liver surgery, there were a large number of studies reporting TOs according to distinct definitions [35]. There was a general concordance in the definitions, as nearly all of them included key criteria, such as absence of high-grade complications, no prolonged hospitalization, no mortality and no readmission. However, discrepancies have been observed in the selection of the cutoff values; for example, in 18 studies, the authors considered hospitalization prolonged if the length of stay exceeded the 75th percentile, while in 10 studies it was the 50th percentile. Analogous discrepancies were related to the no-mortality or no-readmission periods [30 days vs. 90 days] and in the case of the grade of postoperative complications (any grade vs. II grade) as well. Furthermore, other criteria, such as no transfusion, R0 margins or no intraoperative incident, also differed between the studies [35]. Eventually, an expert consensus was developed for the definition of TOs in liver surgery [36]. This example highlights the importance of early definition of the TO in a particular field of surgery, by an international expert consensus, which would allow later a wide application of uniform definition and thus it would make all of the reports comparable.

It should be noted that a multi-institutional expert consensus on TO criteria has been established for liver surgery and also for DIEP flap breast reconstruction, colorectal surgery or emergency laparotomy utilizing a Delphi methodology [36,37,38,39]. In this approach, the objectively selected expert group (panel) proceeds with an anonymous survey. After each round of the survey, the results are analyzed, and a questionnaire for another round is built upon these results, which is again sent to the panel members. These iterations are repeated until the desired consensus is reached [40]. We strongly recommend this method for establishing a multi-institutional expert consensus for the TO definition in any field of surgery.

## 5. Textbook Outcomes in Patients with Skin Malignancies

### 5.1. Textbook Outcomes in Melanoma Patients

In the field of skin malignancies, all TO evaluations were performed in patients with melanoma, with a special focus on patients who underwent surgery after neoadjuvant systemic treatment. Zijlker et al. performed the first analysis of surgery-related morbidity in patients with melanoma, applying the TO as an outcome measure after lymph node dissection [41]. In this preliminary report, with a small sample size, the authors also compared TOs between two groups of patients: one undergoing surgery after neoadjuvant systemic therapy [namely: 6-week combined anti-PD-1 (nivolumab) and anti-CTLA-4 (ipilimumab) immunotherapy] and the other, undergoing upfront surgery without preoperative therapy. The definition of a TO included no reoperation within 30 days, no re-admission and no grade II-V complications (as per the Clavien–Dindo classification) within 90 days, the length of a hospital stay below the 75th percentile and microscopically radical resection (R0). The number of patients who achieved TOs was 50% in the neoadjuvant subgroup and 49% in the patients operated in the initial period, without a significant difference. In particular, the percentages of patients who achieved each specific component of the TO seemed similar in both subgroups, with the avoidance of grade II (or higher) complications being the most limiting factor in both subgroups (69% for neoadjuvant surgery and 63% for initial surgery). Furthermore, the duration of surgery did not differ significantly between the subgroups; however, there was a trend toward a shorter duration of surgery in the upfront operated patients (90 vs. 105 min, *p* = 0.077). It should be noted that, as the authors themselves have also pointed out, this study had several limitations, with the sample size being the most prominent (44 patients in the neoadjuvant subgroup and 76 in the initially operated subgroup) and insufficient for a credible multivariate analysis; thus, the heterogeneity of baseline characteristics between the subgroups could also have influenced the results [41]. Another study conducted by Zijlker et al., which included twice as many patients than in the previous report (treated in other institutions), also focused on comparing the neoadjuvant strategy versus upfront surgery, regarding the surgical outcomes [42]. This time, patients treated with immune checkpoint inhibitors and those who received tyrosine kinase inhibitors (targeted therapy) and a combination of these regimens were included. Besides the group of patients included in this study, the definition of the TO also differed. Contrary to the previous report, this time, the criterion for the length of hospital stay being below the 75th percentile was not applied. Therefore, the reported TO achievement rate was slightly higher than in the previous study: 61% for the neoadjuvant subgroup and 57% for upfront resected patients. Again, in the case of each particular component of the TO, there was no difference depending on the usage of neoadjuvant systemic treatment, and the most limiting factor for the TO achievement was the avoidance of grade ≥ II surgical complications (62% for neoadjuvant-treated and 59% for upfront surgery). In the context of neoadjuvant immunotherapy, which is known for causing immune-related adverse events (irAEs), corticosteroids often are used to alleviate these conditions. Interestingly, the steroid treatment for irAEs has not so far been proven to interfere with the surgical treatment in the neoadjuvant-treated melanoma patients. It has not been observed that the usage of steroids would increase the rate of complications in these groups of patients [41,42], although mechanistically, this kind of treatment could be linked with the impairment of wound healing [43].

### 5.2. Definition of TO in Melanoma Compared to Definitions in Other Cancers

The selection of the TO criteria by Zijlker et al. is concordant with other definitions of the TO, including mainly the key components: no mortality, no grade ≥ II complications, no readmission, no reoperation and R0 margins [41,42]. These components are frequently met in TO definitions for multiple surgical oncology procedures [30,32,35,44]. In Table 1, we present the TO criteria encountered in melanoma, extremity sarcoma and breast cancer studies. The length of hospitalization has been incorporated in one of the reports by Zijlker et al. [41], whereas not in the other [42], and the authors have not provided the rationale for this decision. Inclusion of the length of stay into the TO criteria can be a surrogate indicator of postoperative complications; however, accuracy of this approach is questionable [45]. Moreover, the availability of information about surgical complications makes such surrogate indicators unnecessary. Additionally, the usage of the 75th percentile indicates that approx. 25% of patients will not achieve TOs, just by the definition.

## 6. Impact of the Surgery De-Escalation on the Textbook Outcomes

### 6.1. Melanoma

As the systemic regimens for patients with advanced cutaneous malignancies have become increasingly effective, the question has been raised, whether the extent of surgery can be reduced, in order to decrease the surgical morbidity, while maintaining the benefit of a treatment with curative intention. An example of such a strategy could have been observed in the treatment of patients with locally advanced breast cancer, in whom the tumor reduction after neoadjuvant systemic treatment allows for less extensive breast preservation surgery rather than mastectomy [46]. This approach is considered safe, while improving the patients’ quality of life, and thus it constitutes the standard of care [46,47].

**Figure 4:**
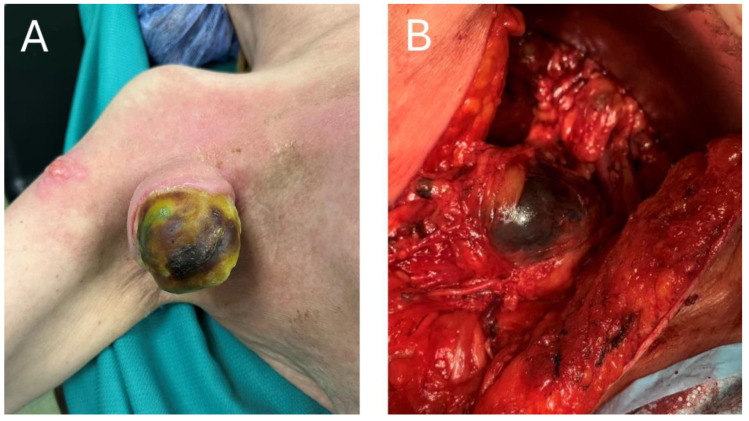
(**A**) Exophytic melanoma tumor after neoadjuvant immunotherapy. (**B**) Intra-operative view of metastatic melanoma tumor post neoadjuvant ICI treatment.

In patients with macroscopic resectable stage III lymph node metastases, therapeutic lymph node dissection (TLND), which has been a mainstay of curative-intent treatment, is very likely to cause postoperative complications and affect the quality of life in those patients [48,49,50]. And although neoadjuvant treatment itself has a great potential to shrink the lesions and facilitate surgery, simultaneously reducing surgical morbidity, there will certainly remain a risk of specific complications, namely the lymphoedema [51,52]. A decrease in the number of TLNDs performed, while maintaining the oncological safety, would be greatly desired, as it would go with a decrease in the number of TLND-induced complications. In particular, given the high prognostic value of a pathological response to neoadjuvant ICI, complete or near-complete responders to these regimens would constitute the most suitable group of patients to de-escalate the surgery [10]. To assess the pathological response without performing a TLND, a very innovative technology, called Index Lymph Node (ILN) resection, has been proposed [12,53,54]. In this approach, at the beginning of a neoadjuvant treatment, the patient undergoes an implantation of a grain-sized magnetic seed into the largest metastatic lymph node. After completion of neoadjuvant treatment, the ILN is supposed to be located using the magnetic probe, removed and assessed for the pathological response. The level of a pathological response can then be used for further therapy guidance: in the PRADO clinical trial, which investigated this strategy, the major pathological responders (0–10% viable melanoma cells in ILN) would proceed directly to follow-up, the partial responders (10–50% viable melanoma cells) would undergo TLND and finally, the non-responders (>50%) would undergo TLND and further adjuvant systemic therapy [12]. In favor of this strategy, the rate of the major pathological responses to the combined neoadjuvant ICI ranges from 35% to 61% [9,10,12,55] and the representativeness of ILN for the rest of the basin reaches 99% [54]. In an optimistic scenario, the number of patients undergoing TLNDs after combined neoadjuvant ICIs could be reduced by approximately 60% if the ILN procedure was implemented. Crucially, this number of patients could be relieved of the complications and consequences of TLND, as ILN resection appears to be much safer and better tolerated than TLND [12]. However, despite these attractive and promising results, the PRADO trial was a phase 2 trial, with insufficient statistical power to conclude that the ILN resection strategy and response-driven therapy are ready for routine clinical practice [12]. More research is warranted, including a phase 3 clinical trial that would confirm these results and possibly change the standard of care for macroscopic stage III melanoma patients. Of note, despite high rates of responses to the neoadjuvant ICI, some patients will still have extensive tumors at the time of surgery, despite neoadjuvant treatment, as is shown in Figure 4.

When considering the TO in the context of de-escalation of the surgery in melanoma patients after neoadjuvant immunotherapy, using the ILN resection instead of TLND, substantial changes to the relevant definition of a TO would be required. The rationale for including the R0 resection margins would be questionable, as the ILN resection by definition omits the whole basin, which would have to be resected to assess the margins. This condition of TO achievement would have to be removed to enable rational TO assessment. On the contrary, it would be useful to capture—other than short-term complications—events related to the removal of lymph nodes and associated with the quality of life, such as lymphoedema.

### 6.2. Basal Cell Carcinoma

In the field of other skin malignancies, in the phase 2 study of patients with advanced basal cell carcinoma treated in the neoadjuvant setting with vismodegib (SSH pathway inhibitor), this agent has shown significant activity in shrinking tumors to facilitate the surgery [56]. The study’s primary endpoint was the downstaging of the tumor; according to the classification of the complexity of the surgical procedure, it needed to remove the tumor. Resection of the tumor required a less complex procedure, if the stage was lower post-treatment. Downstaging was successful in 44/55 (80%) of the patients, supporting the use of the neoadjuvant strategy. Importantly, in 27 patients with a complete clinical response (of whom 25 had the response confirmed by biopsy), 21 patients did not undergo the excision of a scar, reflecting the potential ability to avoid the surgery in cases with favorable response to the systemic treatment. However, unlike the immunotherapy (in which the pathological response produces excellent recurrence-free survival), the patients with complete response to targeted therapy—vismodegib—have frequently developed recurrences (out of 27 patients, 7 had a relapse, while nine were lost to follow-up) [56].

### 6.3. Cutaneous Squamous Cell Carcinoma

Surgical removal of locally advanced CSCC often requires extensive resection, which—especially in the head and neck region, where these tumors commonly occur—causes severe functional or cosmetic impairment [57]. Using highly active systemic regimens, such as ICIs, creates an opportunity to omit or reduce the extent of surgery, which would bring the greatest benefit, especially to patients with bulky tumors, who were initially assigned to an extensive and mutilating surgery [58].

To date, no randomized clinical trials have been reported to evaluate the safety of surgery omission in patients with CSCC who respond to the ICI. However, in the phase 2 clinical trials with neoadjuvant cemiplimab, as well as in a report from a real-world cohort, there are numbers of patients who (after achieving a clinical response or at least stable disease) withdrew a consent for surgery, was reached up to 18.5% [24,26,59]. This suggests that patients may be more interested in maintaining a local control of the disease, rather than undergoing an extensive, mutilating resection. There is also evidence of successful omission of surgery in patients with ICI-treated CSCC, in whom the favorable response to treatment has led to the avoidance of an orbital exenteration [60,61]. Furthermore, as found in the MATISSE trial, those patients who omitted the surgery due to favorable clinical response (in this trial, the response was also confirmed with FDG-PET assessment) had better quality of life measures [59]. Finally, relapses have not been reported in these patients [26,59]; however, to conclude about the oncological safety of this strategy, evidence from randomized clinical trials is warranted. For this purpose, one could imagine a study which would include patients with tumors located in surgically challenging anatomic locations, who achieve favorable response after ICI treatment. The patients would then be randomized to either undergo or omit the surgery. Also, in the VISMONEO clinical trial, in BCC patients, of 55 intention-to-treat patients, 7 withdrew consent for the protocol-specified surgery after achieving clinical response [56].

However, there still remains the question of what method should be used for the evaluation of the response, as it seems clear that a standard RECIST 1.1 assessment underestimates the level of pathological response: in a study by Gross et al. the rate of complete pathological responses was 51%, while only 6% of patients had a complete radiological response [24]. On the contrary, the metabolic response, assessed with the FDG-PET/CT, appears to be more relevant for routine response evaluation in CSCC patients [62,63] and has already been used to confirm the response in patients who withdraw consent for surgery in the MATISSE trial [59]. This suggests that the metabolic response assessment may potentially be used to make the decision to perform or to omit the surgery after ICI treatment in patients who would require extensive and mutilating surgery.

## 7. Conclusions

Novel systemic strategies have major impacts on surgery in skin cancer and are available for patients with locally (or regionally) advanced skin cancers, otherwise referred for vast surgical treatment. With an excellent rate of responses to the neoadjuvant therapy, the surgery can not only be facilitated, but also de-escalated or even omitted in the patients with the most favorable responses confirmed by biopsy—as suggested by preliminary evidence. In selected clinical situations, the balance between risk and benefit may favor disease control achieved with systemic treatment over the definitive, but extensive surgery. In order to comprehensively assess the expected surgical morbidity, as well as oncological safety, a novel outcome measure has been introduced—the Textbook Outcome. It can be easily adjusted to different surgical procedures, including those used for treatment of patients with skin malignancies. In the context of melanoma patients, the TO has already been used to show that the neoadjuvant systemic treatment does not aggravate surgical morbidity. On the field of skin malignancies, further research is warranted to establish the role of TOs in guiding clinical decisions or in providing prognostic information.

However, in order to prevent the discrepancies in the definition of TOs among distinct studies, an expert consensus should be established. For that purpose, we recommend the Delphi methodology.

## Figures and Tables

**Table 1:** Comparison of Textbook Outcome definitions among different neoplasms. ICI—immune checkpoint inhibitors; TT—targeted therapy, TO—Textbook Outcome. *—Aitken et al. presented data for chemotherapy, hormonal and radiation therapy, however it is not indicated whether these were given prior to the surgery.

	Zijlker et al., 2023 [41]		Zijker et al., 2024 [42]		Lazarides et al., 2020 [30]	Aitken et al., 2022 [32]
Disease	Stage III melanoma		Stage III melanoma		Soft tissue sarcoma of the extremities	Breast cancer
Number of patients	44	76	89	79	7658	75,063
Neoadjuvant treatment	ICI	None	ICI/TT/ICI + TT (in 29/16/44 patients)	None	Radiation (18.1%), no data for chemotherapy	Unknown *
Rate of TO	50%	49%	61%	57%	56%	40.8%
**Criteria for TO:**						
R0 margins	+		+		+	+
Length of stay <75th percentile	+		-		+	+(<50th percentile)
No 90 d readmission	+		+		+(30 d)	+(30 d)
No 90 d grade II–V complications	+		+		-	-
No reoperation within 30 d	+		+		-	-
No mortality	-		-		+(90 d)	+(30 d)
Guidelines-compliant lymphadenectomy	-		-		-	+(≥10 nodes)

## Data Availability

The original contributions presented in the study are included in the article, further inquiries can be directed to the corresponding author.
